# Understanding Seasonal Changes to Improve Good Practices in Livestock Management

**DOI:** 10.3389/fpubh.2018.00175

**Published:** 2018-06-15

**Authors:** Francesco Martelli, Claudia Giacomozzi, Antonello Fadda, Chiara Frazzoli

**Affiliations:** Department of Cardiovascular, Dysmetabolic and Aging-Associated Diseases, Istituto Superiore di Sanità, Rome, Italy

**Keywords:** dairy chain, cow milk, seasonality, risk management, risk assessment, food safety, livestock management, One Health

## Abstract

**Background and Aim:** Food quality control techniques based on process control methods are increasingly adopted in livestock production systems to fulfill increasing market's expectations toward competitiveness and issues linked to One Health pillars (environment, animal, and human health). Control Charts allow monitoring and systematic investigation of sources of variability in dairy production parameters. These parameters, however, may be affected by seasonal variations that render impractical, biased or ineffective the use statistical control charts. A possible approach to this problem is to adapt seasonal adjustment methods used for the analysis of economic and demographic seasonal time series. The aim of the present work is to evaluate a seasonal decomposition technique called X-11 on milk parameters routinely collected also in small farms (fat, protein, and lactose content, solids-not-fat, freezing point, somatic cell count, total bacterial count) and to test the efficacy of different seasonal removal methods to improve the effectiveness of statistical control charting.

**Method:** Data collection was carried out for 3 years on routinely monitored bulk tank milk parameters of a small farm. Seasonality presence was statistically assessed on milk parameters and, for those parameters showing seasonality, control charts for individuals were applied on raw data, on X-11 seasonally adjusted data, and on data smoothed with a symmetric moving average filter. Correlation of seasonally influenced parameters with daily mean temperature was investigated.

**Results:** Presence of seasonality in milk parameters was statistically assessed for fat, protein, and solids-non-fat components. The X-11 seasonally-adjusted control charts showed a reduced number of violations (false alarms) with respect to non-seasonally adjusted control chart (from 5 to 1 violation for fat, from 17 to 1 violation for protein, and from 9 to none violation for solids-non-fat.). This result was achieved despite stricter control chart limits: with respect to raw data charts, the interval of control chart allowed variation (UCL–LCL) was reduced by 43% for fat, by 33.1% for protein, and by 14.3% for solids-not-fat.

**Conclusions:** X-11 deseasonalization of routinely collected milk parameters was found to be an effective method to improve control chart application effectiveness in farms and milk collecting centers.

## Introduction

Food security, including safety, from livestock systems is of highest importance in human nutrition and one of the multifaceted aspects of sustainability (1, 2). The breeding sector is regulated by economic, political, as well as socio-demographic drivers that, in their turns, cannot ignore sustainability issues linked to One Health pillars (environment, animal, and human health) and their interconnections.

Given the peculiarities of food production chains (usually entailing highly perishable products, low or large batches volumes, great variability in raw materials characteristics, processing, and distribution) a significant effort has been devoted to increase and guarantee the general quality of finished products using industrial management practices covering the whole production chain (3, 4). In the last decades food industry has been pushed to implement a wide range of food quality management protocols to reply the increasing consumers' expectations, especially following major food crises (4), environmental alerts, globalization of markets of food products and food producing animals, globalization of dietary habits (1, 5), and upset of toxicant related zoonoses (6, 7).

In the precision dairy farming era, food quality control techniques based on process control methods and quality improvement programs are gaining increasing attention. In fact, environmental factors (from essential nutrients to toxic contaminants and agro-zootechnical residues) at the environment-animal-human interfaces impact severely on food security and food safety (7, 8), with impact on current and next generation (8).

Close monitoring of all factors implied in food chains management allows early management of anomalous events, thus leading to a general increase in food quality and safety, general enterprise's competitiveness (demonstrable deep quality control) and profitability (including decreased risk of undesired events and subsequent food waste and losses) along with gain in environmental sustainability (9–12).

Process monitoring techniques are based in the strict monitoring of sources of variability in any production phase. Systematic investigation on the root causes of any unusual source of variability, together with variability reduction techniques are the pillars of process control methods.

In the last decade, several attempts have been done to apply Statistical Process Control (SPC) in dairy production systems and in general livestock management (13), mostly based on traditional Shewart control chart (or Cusum control chart) (14) The relevance of some studies, at least from the point of view of practical benefits, is somehow unclear [for dairy herd, see (15)].

Regardless of the specific SPC implementation, most of the process monitoring techniques aim at the separation of the overall variation in a routine variability (also known as “chance causes”) and an exceptional variation (or “assignable cause”) originating from a change in the process that would be worth analyzing (16). If the chance causes variability magnitude is comparable to the variability due to assignable causes, the task of extracting a meaningful alert signal indicating the need of intervention on the process can be compared to that of extracting a meaningful signal from measurements extremely corrupted by noise. An example of chance causes in dairy production can be the normal biological variation in milk composition, while assignable causes can derive from animal illness, feeding, unplanned variations in herd management, or their consequences.

In addition, dairy production parameters routinely collected both in farms and Milk Collecting Centers may be affected by seasonal variations that render impractical or ineffective the use of some of SPC techniques, like statistical control charts, which are typically based on the underlying assumptions of independence and stationarity of observations (16, 17).

A possible approach to this problem is to use or adapt seasonal adjustment methods routinely used by national statistical offices and central banks, whose work is frequently based on analysis of economic and demographic seasonal time series.

Between those techniques, an entire category of non-parametric methods has been developed starting in the 60's (18, 19) to decompose time series into unobservable components using iterative procedure based on successive filtering, such as the X-11 family of methods (X-11, X-11-ARIMA, X-12-ARIMA). The X-11 method was introduced in 1965 by the United States Census Bureau as practical tool for seasonal decomposition of time series. X-11 uses an iterative approach to estimate the components of a time series. At each step different moving averages filters are used to decompose the time series into a trend/cycle component (a long term evolution/a slow movement around the trend), a seasonal component (Intra-year variations repeating regularly year after year), and an irregular component (Random fluctuations).

The Seasonal component should represent fluctuations in the data recurring with the same pattern, intensity and timing. In certain models, a modification in the seasonal component over the years timeline can be coped for to represent long term changes which gradually evolve as a response of a global, systemic change. In the former case, a stable seasonality is present in the time series, while in the latter a moving seasonality is said to be present.

The Trend or Cycle component takes in account a steady tendency (trend of growth, or decline) over a significantly long period of time; sometimes another component, generally alternating over a period of time greater than the year, may be superimposed over the trend and is generally called Cycle component.

The Irregular component is what remains of the time series after adjustment for seasonality and trend. It should represent mainly measurement errors, calendar changes, or exceptional events which cannot be forecast and have a significant influence on the time series.

Different models have been proposed over time to model the influence of each component in the total variation represented in the time series. Basically, additive models assumes that the magnitude of the components are independent from each other; multiplicative models assume that all three components are dependent on each other; finally, pseudo additive models assume the independence of S and I, but the dependence of S and I from C (20).

Seasonality adjustment is increasingly considered as a useful tool in livestock management (21–24), under the pressure for improving general efficiency and consumer acceptance, reducing waste, and increasing trading margins.

Another growing application for seasonality adjustment is the regulatory area: seasonality adjustment is one of the adjustment techniques adopted by the Irish national Department of Agriculture, Food and the Marine (25) in their calculations over bulk tank somatic cell counts requested by EU Regulation 853/2004 (26).

The main aim of this work is to conduct an evaluation of basic X-11 seasonal decomposition technique on data routinely collected in small farms (fat content, protein content, lactose content, solids-not-fat, freezing point, somatic cell count, total bacterial count) and to test the efficacy of seasonal removal methods to improve the impact of statistical control charting.

As a case study, we provide an application example on data coming from a 3 year long measurement campaign on a small dairy farm.

## Materials and methods

### Data collection and management

Data collection was carried on between January, 2011 and December, 2013 within the framework of the ALERT project^1^ for the monitoring of wholesomeness and quality in the cow milk chain from primary (dairy farm) to secondary production (transformation industry). During this time span, data were collected from raw milk production of a small farm (in the following, EP).

The dairy farm was representative of a well-conducted, medium-sized dairy farm of Central Italy (27). All diagnostics were carried on at the Istituto Zooprofilattico Sperimentale delle Regioni Lazio e Toscana (IZSLT), a public body operating in the frame of National Health Service with duties related to animal health and welfare and food safety.

EP milk production was sampled three times for month (mean inter sample day span = 9.85 days, *SD* = 3.01). A total of 110 samples were acquired and analyzed during the study period. Milk samples were refrigerated at 4 (±2)°C and carried to the testing facilities of IZSLT. Raw milk samples were tested for fat content % (Fat), protein content % (Protein), lactose content % (Lactose), solids-not-fat % (SNF) (all % by weight), freezing point (m°C), somatic cell count (SCC, x1000 cfu/mL), total bacterial count (TBC x1000 cfu/mL).

All lab analyses were carried on the samples within an average of 3.18 days (*SD* = 2.28) from sample collection. All parameters were analyzed following accredited IZSLT testing methodologies described in Table 1.

**Table 1:** Sample analysis methods.

**Parameter**	**IZSLT method/internal reference**
Total bacterial count	Fluoro-opto-electronic method (POS CIP 021 INT rev 3 2010)†
Somatic cell count	Fluoro-opto-electronic method (POS CIP 018 INT rev 5 2009)^§^
Fat, lactose, protein content; freezing point	IR Spectrophotometry (POS CIP 018 INT rev 5 2009)^§^
SNF	Gravimetric analysis (Rapporti ISTISAN 1996/34, pp. 7–10, Met B)

† *Updated to rev 4 on 2013-02-01*.

§ *Updated to rev 6 on 2012-03-01 and to rev 8 on 2013-02-01*.

All data coming from the data collection procedures were imported into a purposely designed relational database at Istituto Superiore di Sanità (ISS) facilities. All subsequent analyses were carried on extrapolating raw data from this database. Additional info on the environmental temperature for the whole sampling period was added in the database. Climate data (daily mean temperature) was gathered from the official Istituto superiore per la protezione e la ricerca ambientale (ISPRA) database (28), at the closest monitoring station (~8 Km from the EP farm).

The presence of seasonality in data parameters was initially assessed by visual inspection in raw data.

A more detailed seasonality test was carried on converting the raw data points into a 36 point monthly series (all samples from the same month were averaged, resulting in a 36 point data series) and by execution of Friedman test and Kruskal–Wallis test on monthly averaged data.

A *p-*value lower than 5% was the limit set to reject the null hypothesis of no seasonal effect.

### Control chart analysis

Parameters coming from EP raw milk production showing a marked seasonal effect (Fat, Protein, SNF) were analyzed using control chart for individuals with three alternative approaches:

#### Method A: control chart for individuals using raw data

Control chart for individuals were plotted using raw data.

#### Method B: control chart for individuals using X-11 seasonally adjusted data

Monthly time series were adjusted for seasonality using JDemetra+, X-11 additive method (18).

The algorithm used can be described as follows:

1. derive an initial estimate of the trend-cycle TC1 by applying A symmetric moving average moving average to the raw data;
2. subtract this estimate from the original time series in order to get an initial estimate of the seasonal-irregular (SI) component;
3. apply a moving average to the SI to obtain an initial estimate of the seasonal component S1;
4. subtract the initial S1 component from the raw data to obtain an initial estimate of the seasonally adjusted series SA1 (i.e., the trend-cycle/irregular);
5. apply a Henderson moving average to obtain a second estimate of the trend-cycle TC2;
6. subtract TC2 from the raw data to obtain a second estimate of the SI (SI2), and apply a moving average to obtain final estimates of the seasonal component (S);
7. subtract S from the raw data to obtain a final estimate of the seasonally adjusted series (SA2) and apply a Henderson moving average to obtain a final estimate of the trend-cycle TC;
8. subtract S from the SI2 to obtain an estimate of the irregular component (I).

Trading days and Easter effect were neglected. Control chart for individuals were constructed in Matlab using the seasonally adjusted time series.

#### Method C: control chart for individuals using moving average seasonally adjusted data

Raw data was smoothed using moving average Whittaker–Henderson 13-term filter (29, 30) in order to get a gross approximation of seasonal component (GSC). A season-adjusted time series was derived subtracting the smoothed data from the raw data. Control chart for individuals were constructed in Matlab on the time series obtained subtracting the GSC from the raw data.

For all control charts Upper and Lower Control limits (UCL and LCL) were calculated using the following relationships

$$\begin{array}{l} UCL\;=\;\mu_{p}+3^{*}\sigma_{p}\\ LCL\;=\;\mu_{p}-3^{*}\sigma_{p} \end{array}$$

Where

μ_p_: Estimated process mean;

σ_p_: Estimated process standard deviation

Both estimated process parameters were calculated using the Matlab's control chart implementation.

Performance of the three algorithms was compared visually examining the identified trends (where available), and comparing the resulting estimated process means, standard deviations, range, Upper and Lower Control Limits, and number of process violations.

All correlational and statistical analyses were carried on in Matlab (The MathWorks, Inc., Natick, Massachusetts, USA), directly interfacing the relational database. For specific topics, data extracted from the database were analyzed using statistical package R (31), and JDemetra+^2^ (rel. 2.2.0). Wherever applicable, all seasonality analysis were carried on following the European Statistical System (ESS) guidelines on seasonal adjustment (32).

## Results

### Preliminary analysis

As a general outlook, milk data showed both seasonally variable and seasonally stable parameters.

**Figure 1:**
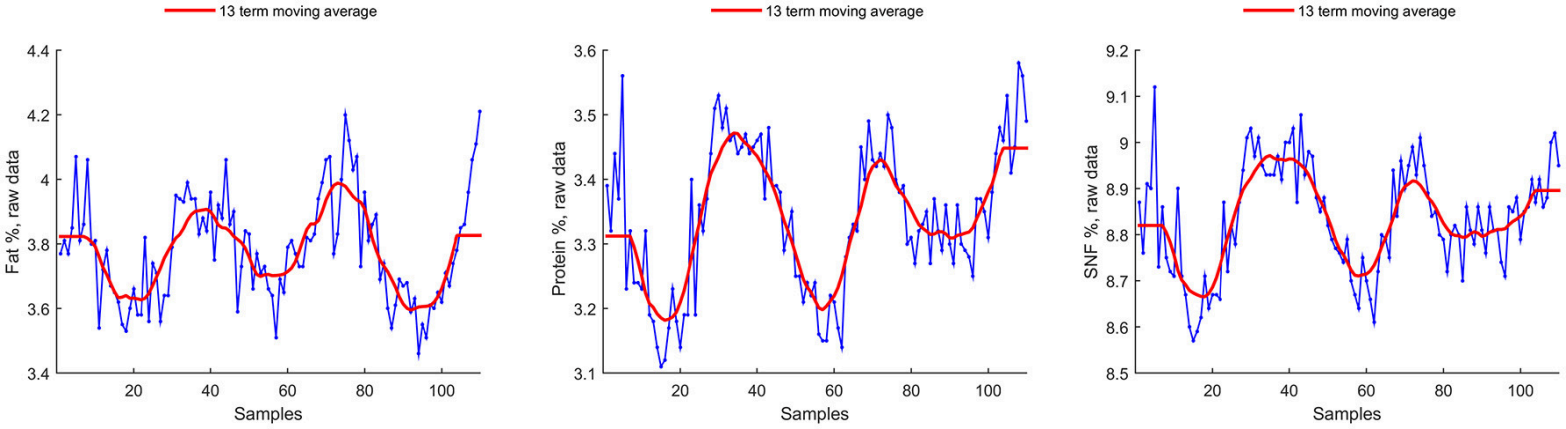
Seasonal parameters raw data, with superimposed Witthaker–Henderson filtering (Method C).

Visual inspection reveals a seasonal effect on fat %, protein %, and solid non-fat % (Figure 1). A red trend line (method C) was superimposed to facilitate the identification of the general trend.

**Figure 2:**
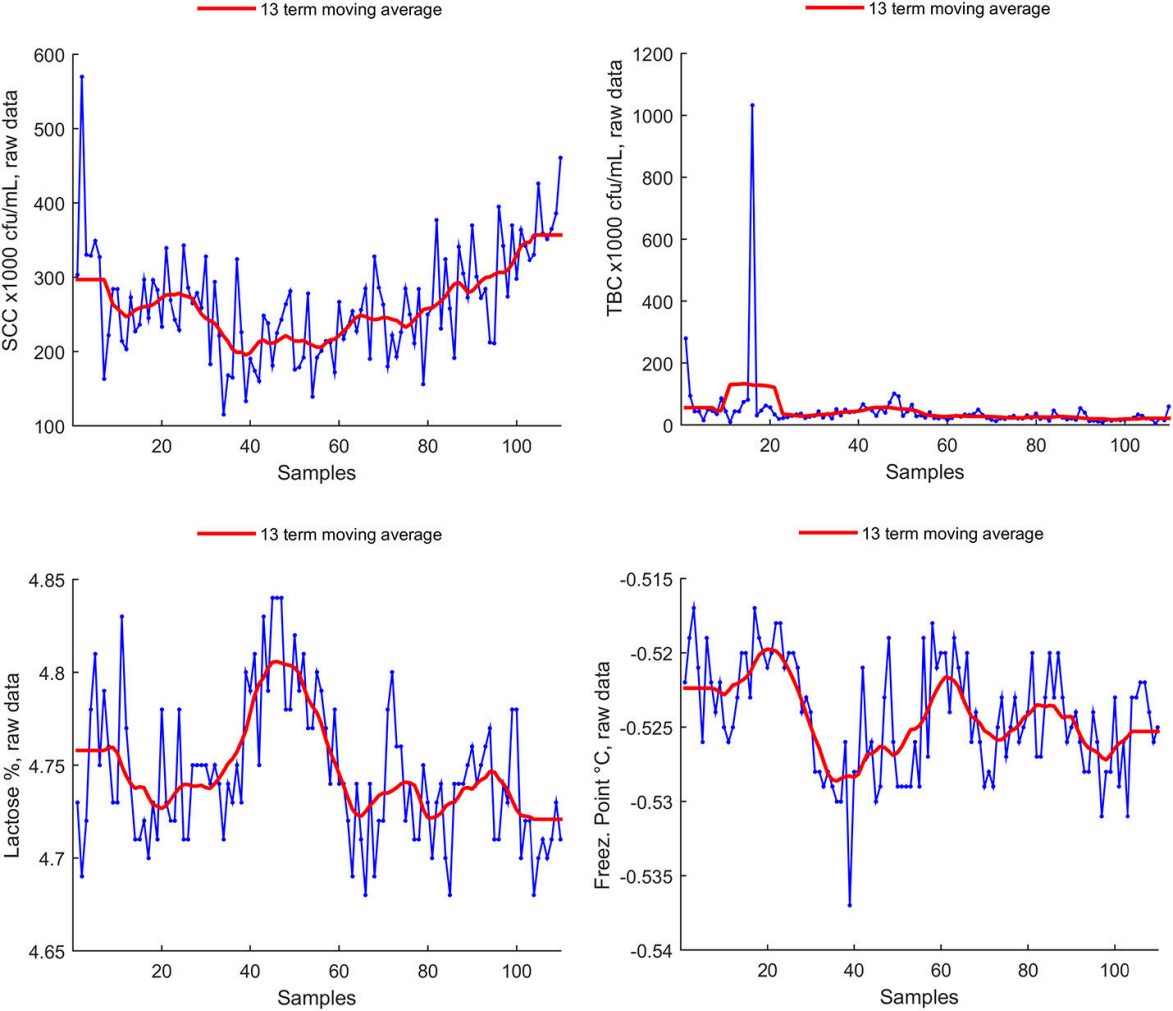
Non-seasonal parameters raw data, with superimposed Witthaker–Henderson filtering (Method C).

Freezing point seasonality is unclear by visual inspection, as well for somatic cell count, and lactose (Figure 2). A clear outlier is present in total bacterial count.

Descriptive statistics (Mean, standard deviation *SD*, Range, Minimum and Maximum) for the original time series and for the monthly time series are reported in Table 2.

**Table 2:** Descriptive statistics of original data.

		***N***	**Mean**	***SD***	**Range**	**Min**	**Max**
Original time series	Fat %	110	3.7831	0.1659	0.7500	3.4600	4.2100
	Protein %	110	3.3406	0.1142	0.4700	3.1100	3.5800
	SNF %	110	8.8336	0.1176	0.5500	8.5700	9.1200
Monthly time series	Fat %	36	3.7868	0.1478	0.5892	3.5375	4.1267
	Protein %	36	3.3389	0.1073	0.4000	3.1433	3.5433
	SNF %	36	8.8312	0.1057	0.3787	8.6133	8.9920

### Statistical analysis for seasonality

Statistical analysis (JDemetra+) confirmed the presence of seasonality effect on fat, protein, and solid non-fat; there was no confirmed seasonality for freezing point, somatic cell count, total bacteria count, and lactose. For TBC, no evidence of seasonality was present even removing the clear outlier present. Results of this analysis are reported in Table 3.

**Table 3:** Statistical seasonality assessment in raw data.

	**Friedman test**		**Kruskall–Wallis test**	
	***F***	***p***	***H***	***p***
Protein	29.8718	**0.0017**	31.7087	**0.0008**
Fat	29.0000	**0.0023**	30.3273	**0.0014**
Lactose	16.9487	0.1094	18.0150	0.0812
SNF	29.4615	**0.0019**	30.5195	**0.0013**
Freez. Point	14.6923	0.1970	16.2192	0.1332
SCC	9.5128	0.5747	10.3333	0.5007
TBC	19.6667	0.0501	18.4294	0.0721

*In bold, the significant values (p < 0.05)*.

### Control chart analysis

#### Method A

**Figure 3:**
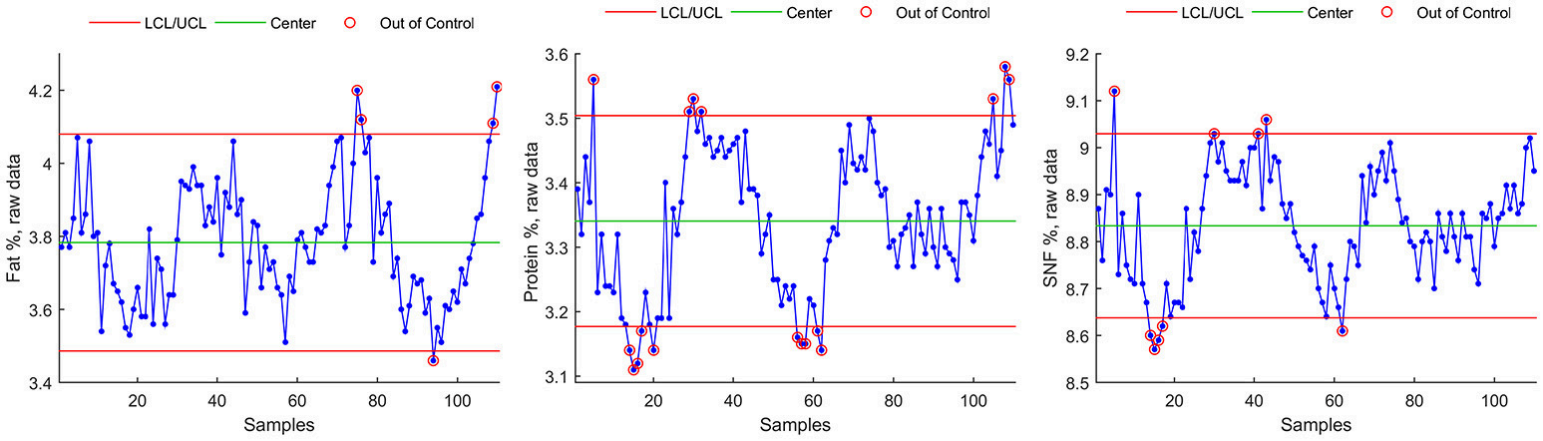
Control Chart for individuals for raw data (Fat, Protein, SNF).

Control chart for individuals were plotted on raw data and are shown in Figure 3 for Fat %, Protein %, and SNF %. Main numerical results of Method A can be found, for each milk component, in Table 4.

**Table 4:** Control Chart estimated process mean μ_p_ and standard deviation σ_p_, range, Upper and Lower Control Limits and number (#) of process violations for the three methods.

		**μ_p_**	**σ_p_**	**Range [Min Max]**	**UCL**	**LCL**	**# Violations**
**Method A**	Fat %	3.783	0.099	0.75 [3.46 4.21]	4.08	3.49	5
	Protein %	3.341	0.055	0.47 [3.11 3.58]	3.50	3.18	17
	SNF %	8.834	0.065	0.55 [8.57 9.12]	9.03	8.64	9
**Method B**	Fat %	3.787	0.056	0.33 [3.67 4.00]	3.96	3.62	1
	Protein %	3.339	0.036	0.22 [3.22 3.44]	3.45	3.23	1
	SNF %	8.831	0.056	0.25 [8.70 8.95]	9.00	8.66	0
**Method C**	Fat %	0.008	0.095	0.61 [−0.22 0.38]	0.29	−0.28	1
	Protein %	0.005	0.050	0.36 [−0.12 0.25]	0.15	−0.15	1
	SNF %	0.004	0.059	0.42 [−0.12 0.30]	0.18	−0.17	1

#### Method B

Seasonal analysis on monthly time series (MTS) resulted in four time series, representing the seasonal component (S), the irregular component (IRR), the trend (T) component, and a seasonal-adjusted component (SA). Under the additive modality of analysis, the following relationships stand true:

(1) $$\begin{array}{r} MTS=S+IRR+T \end{array}$$

(2) $$\begin{array}{r} SA=\;IRR+T=MTS-S \end{array}$$

**Figure 4:**
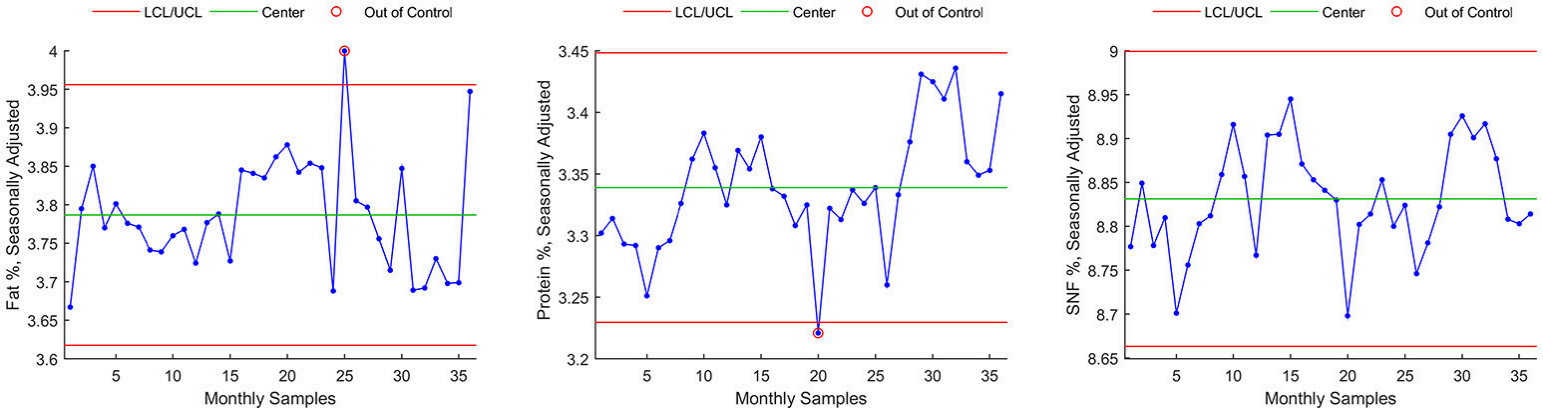
Control chart for individuals for seasonally adjusted series.

Control chart for individuals for seasonally adjusted series (SA) are shown in Figure 4 for Fat %, Protein %, and SNF %.

The JDemetra+ package outputs an overall measure of quality of decomposition called Q statistic, whose value is considered satisfactory if less than unity. Q statistics for Fat %, Protein %, and SNF % were, respectively, 0.55, 0.42, and 0.65.

**Figure 5:**
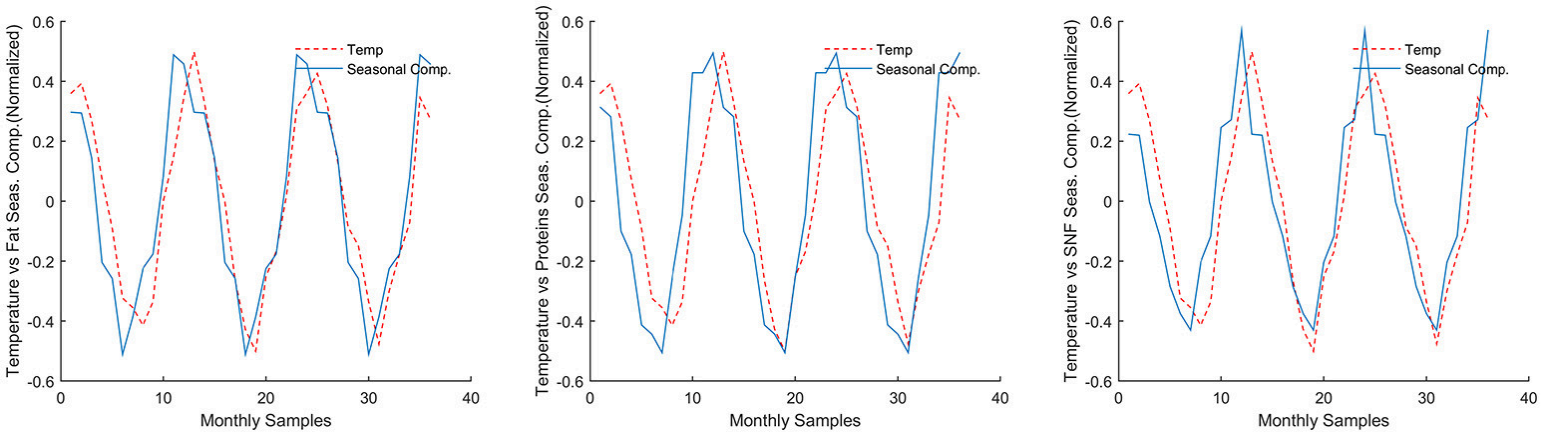
Correlation of seasonal components with daily mean temperature. Both data mean-normalized, temperature y axis is inverted.

Another indirect evaluation of the quality of decomposition is the negative correlation between Fat, Protein, and SNF seasonal components (S) and daily mean temperature, as shown in Figure 5.

Pearson correlation coefficients and their associated *p-*levels are reported in Table 5.

**Table 5:** Pearson correlation coefficients between daily mean temperature and Fat, Protein, and SNF seasonal components.

**Daily mean temperature vs:**	***r***	**[95% CI]**
**Fat %**	−0.90^**^	[−0.82 −0.95]
**Protein %**	−0.81^**^	[−0.65 −0.89]
**SNF %**	−0.85^**^	[−0.73 −0.92]

** *p < 0.001*.

**Figure 6:**
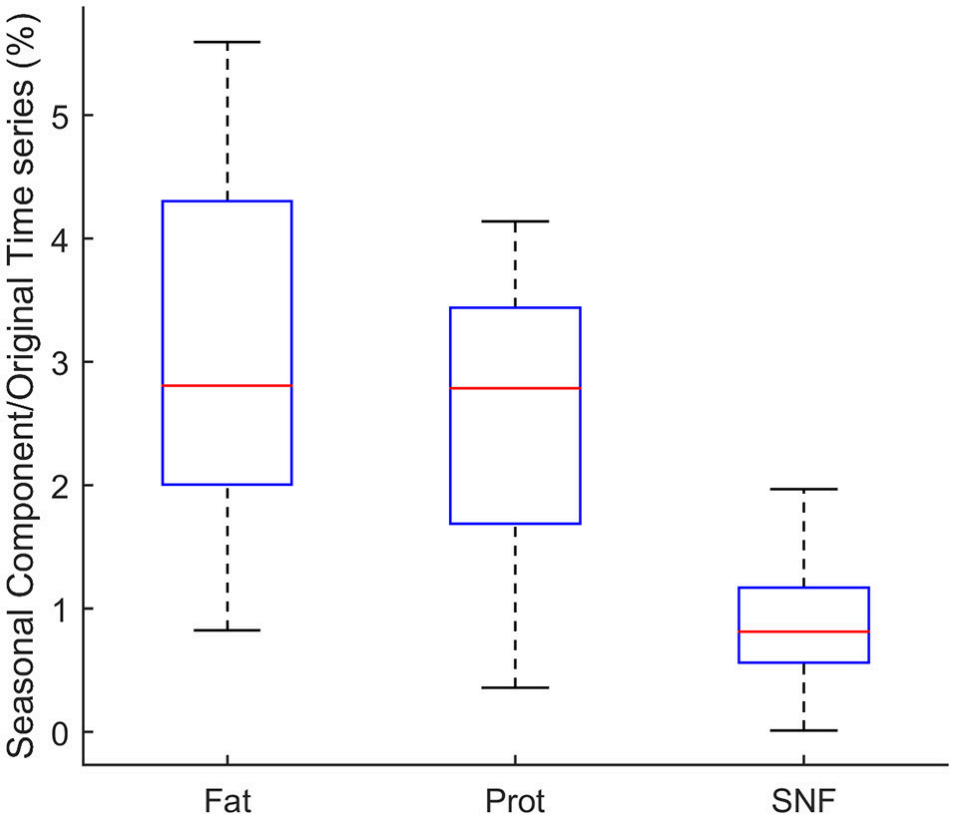
Relative (%) contribution of the seasonal components (S) to the overall time series.

An estimation of the relative (%) contribution of the seasonal component (S) to the overall time series MTS (Equation 1) is given in Figure 6 for fat, protein, and SNF.

As shown in the figure, the seasonal component for Fat % is in average about 2.81% of the MTS series. For Protein % and SNF % the seasonal component accounts respectively for 2.79 and 0.81% of the MTS.

Main numerical results of Method B seasonal adjustment can be found, for each milk component, in Table 4:

- For Fat component, Method B seasonal adjustment led to a reduction of control chart violations from 5 to 1 (Figures 4, **8**, rectangles). The data Range [max(Fat)–min(Fat)] was reduced from 0.75 to 0.33, and the interval of control chart allowed variation (UCL–LCL) was almost halved (from 0.59 to 0.34) with respect from raw data control chart (Method A), corresponding to a 42.3% reduction.
- For Protein component, Method B seasonal adjustment led to a reduction of control chart violations from 17 to 1. The data Range [max(Protein)–min(Protein)] was reduced from 0.47 to 0.22, and the interval of control chart allowed variation (UCL–LCL) was reduced from 0.32 to 0.22) with respect from raw data control chart (Method A), corresponding to a 31.3% reduction.
- For SNF component, Method B seasonal adjustment led to the absence of control chart violations (from 9 to 0). The data Range [max(SNF)–min(SNF)] was reduced from 0.45 to 0.25, and the interval of control chart allowed variation (UCL–LCL) was reduced from 0.39 to 0.34 with respect from raw data control chart (Method A), corresponding to a 12.8% reduction.

#### Method C

In this method, a smoothed time series is subtracted from raw data, in order to get an estimate of the variability of the time series not due to seasonal variation.

Smoothing is achieved using a 13 term Henderson filter, which is a symmetric moving average type filter designed to let annual trends to pass unchanged through the filter. The smoothed time series, resulting from the filter action is shown in red in Figures 1, 2.

**Figure 7:**
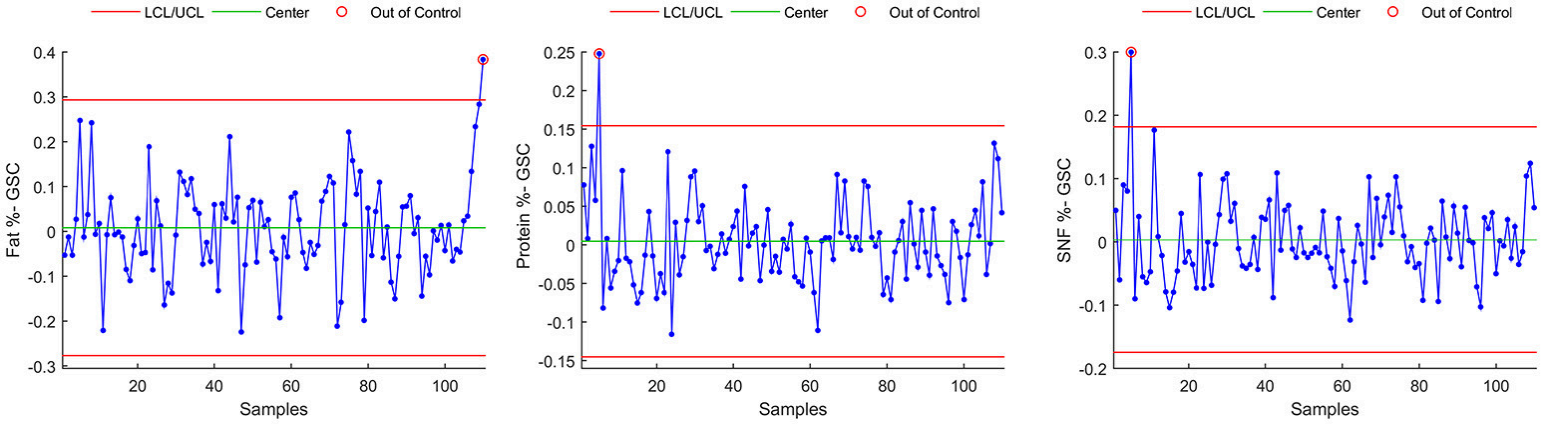
Control chart for individuals on the H13 smoothed time series.

Control chart for individuals on the resulting time series data are shown in Figure 7 for Fat %, Protein %, and SNF %. Main numerical results of Method C can be found, for each milk component, in Table 4.

### Final results and comparison of methods

For each of the three methods, estimated process mean and standard deviation, range and Upper and Lower Control Limits for the resulting time series are given in Table 4, together with the number of data points exceeding lower or upper control limits (violations).

**Figure 8:**
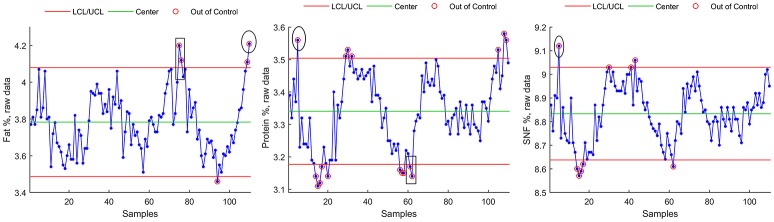
Raw data control charts with Method A (small circles), Meth B (rectangles) and C (ellipses) violations.

Finally, a data plot showing Fat, Protein, and SNF raw data control chart highlighting UCL and LCL violations detected by the three methods is shown in Figure 8.

## Discussion

The current study focused on the application of control charts, a statistical process control technique, to seasonally influenced bovine milk parameter routinely collected from raw milk production of a small farm. While the application of control charts in herd management has been already advocated (13–15), literature on successful applications of control charts to monitor and manage trends in animal production systems is still relatively scarce, and results clearly demonstrating practical benefits are still lacking (13). As pointed out by other authors (14), autocorrelation of time series resulting from seasonality of the observed parameters complicates the application of control charts in biologically derived time series. Another relevant obstacle for the application of any statistical process control technique is the presence of missing data, either derived by technical glitches or by loose management techniques. In this study, which encompassed three complete years, careful planning of data collection procedures led to a complete dataset of 110 measurements without any missing data. Seasonality presence, usually investigated through linear ANOVA models (33), was assessed in method B using Friedman's test on monthly averaged data, which, being non parametric, does not need normality assumption, a condition than can be unmet in practice, and which is usually addressed through logarithmic transformations (23). This study's choice, while being relatively irrelevant for the implementation of control chart techniques—which are considered to be robust to deviation from normality (16)—may however represent in advantage in assessing the presence (or absence) of seasonality on collected milk parameters. In our study, seasonality was found in fat, protein, and SNF components of raw bovine milk, thus corroborating previous studies. Regarding fat and protein components, in fact, there is a general accordance on the presence of seasonality (34–38). For SNF, our study assessed seasonality not confirmed by other authors (33, 34), even though both cited papers reported statistically significant increase of SNF component in early autumn.

We could not assess statistically significant seasonality for lactose, somatic cell count, total bacterial count and freezing point parameters. While we did not found sufficient literature on freezing point seasonality, lactose content seasonality is still somehow debated [see (33, 35) for presence of seasonality, and (34, 36, 37) for unclear presence or absence of seasonal effects on lactose content], as well as total bacterial count seasonality [(23, 35, 38) for presence—(33), for absence]. We found no seasonal effect on somatic cell count, despite prevalent literature consistently reports on seasonality (33, 34, 36–38), an exception being (35). Our data (Figure 2) show both a trend and several spikes, but—quite unexpected—no evidence of cyclic patterns. Investigation on this aspect is still ongoing.

The application of the X-11 algorithm (Method B) asked for monthly averaging of collected data, which can be a drawback because of the inherent loss of information deriving from the averaging process. This choice could be a limitation, since the amount of raw data collected for the study was considerably bigger than in previous studies (33, 34, 36). However, we could demonstrate a relevant reduction of the number of control chart limits violation on seasonally adjusted data, in comparison with the application of the same technique on raw data (Method A); this reduction was achieved, given the additive model used, by subtracting from the original data a seasonal component which accounts for (Figure 6) only a few percent of the raw time series. This observation summarizes that the total effect of both the averaging process and the deseasoning method on the amount of information present in the raw time series could be considered somehow limited.

It must be noted that the reduction in number of violations of control chart limits has been achieved despite a marked reduction (bigger for fat component, smaller for SNF component) of the interval of allowed variation (ULC–LCL). As a consequence, seasonally adjusted control charts could be more suitable than raw data control charts in revealing sudden deviations of in bulk milk components.

The proposed seasonal adjustment process (Method B) in statistical control charting could be of interest for additional reasons, besides the removal of parameter's seasonality. Following a general decomposition model, X-11 method decomposes the observed time series in three fundamental components, namely Seasonal (S), Trend or Cycle (T or C), and Irregular (I). The Seasonal component should isolate the periodic pattern, while the Trend component should contain linear or nonlinear long-term trends, and cycles with periodicity greater than the Seasonal period. The irregular component is usually defined as the cumulative component of all unpredictable effects and sampling errors. This decomposition could be of interest in dairy production systems. As an example, the isolated seasonal component (S) in both Fat, Protein, and SNF time series showed a strong correlation with daily mean temperature, thus corroborating previous works (33–37). Trend and Cycle components could be subject to further analysis, in order to investigate correlations with herd management techniques, or general animal's health status.

In this study, a reduction in control limit violations is obtained also through Method C. This method has a simple implementation but it showed to be ineffective in detecting parameter's shifts that are easily detected by both methods A and B (Figure 8, rectangular areas). Method C also showed sensitivity to outliers and time series extremes. This latter aspect is due to the symmetry of the applied Henderson filter whose performances degrades, by construction, at the beginning and at the end of the time series.

### Interpretation and relevance of study findings

In the social and economic contexts, seasonal adjustment is often used to remove the seasonal component from time series, mostly because it can be a confounding factor for movements in other components of greater economic significance (20). Similarly, the Irregular component is seen mostly as background noise, deriving from sampling errors or unpredictable events.

A major distinguishing factor in the application of seasonal adjustment in farming industry is that all seasonal, trend, cycle, and irregular components may be of interest.

In the food/farming industry, evidence suggests that a slightly different interpretation of the relevance of the three components should be adopted. While it is certainly true that the removal of the seasonal component may reveal hidden trends, it should be noted that this component is a manifestation of a biologically and physiologically relevant process. For this reason the seasonal component may itself contain valuable information on animal health, and any intervention leading to its modification could be of economical relevance.

The irregular component, which represents both the background noise of the process but also the effects of sudden changes in biological processes, may be of extreme interest in all those contexts where strict temporal monitoring of dynamically evolving parameters may be a driver of quick corrective intervention on animal's health and wellbeing, covering nutrition and herd management in general.

As an additional remark, some relevant topics in classical applications of seasonality adjustment may not be useful in milk production systems: for instance, trading days and holidays effects, which are usually taken into account in a socioeconomic analysis, may be of little relevance, since milk production process is primarily influenced by herd physiology and natural effects. Trading days, holidays/Easter effects could arise only indirectly from animal management (feeding, milking). However, in the farm involved in the study, all animal management activities are carried on in the same way every day, 365 days per year.

The study confirmed the correlation between Fat %, Protein %, SNF %, and environmental temperature. While this finding does not offer, in line of principle, new insight on seasonally sensitive parameters in respect to what can be found on available literature, the correlation strength may suggest that the seasonal component could be used as monitoring parameter in dairy herd management. Seasonally biologically sensitive processes, in fact, are influenced by herd management (e.g., feeding) that can have an impact on the seasonal components of the time series and, indirectly, on the nutritional composition of raw milk. Reshaping seasonality by feeding and other good practices (39), however, need further confirmation and deserves further applied research.

The present work, in terms of statistical control charts, is a phase I study, where historical data are used to construct control limits; these limits are being applied in an ongoing phase II study.

## Author contributions

FM and CF conceived and planned the research, planned and performed data acquisition and analysis. FM, CG, and CF contributed to the interpretation of the results. FM and CG took the lead in writing the manuscript. All authors provided critical feedback and helped shape the research, analysis and manuscript drafting and revising critically the work and approved the final version to be published.

### Conflict of interest statement

The authors declare that the research was conducted in the absence of any commercial or financial relationships that could be construed as a potential conflict of interest.

## Footnotes

^1^www.alert2015.it

^2^https://ec.europa.eu/eurostat/cros/content/software-jdemetra_en) (JDemetra is a software tool officially recommended for the seasonal and calendar adjustment of official statistics in the EU).
